# Does a Plant-Based Diet Stand Out for Its Favorable Composition for Heart Health? Dietary Intake Data from a Randomized Controlled Trial

**DOI:** 10.3390/nu14214597

**Published:** 2022-11-01

**Authors:** Justina Dressler, Maximilian Andreas Storz, Carolin Müller, Farid I. Kandil, Christian S. Kessler, Andreas Michalsen, Michael Jeitler

**Affiliations:** 1 Institute of Social Medicine, Epidemiology and Health Economics, Charité–Universitätsmedizin Berlin, Corporate Member of Freie Universität Berlin and Humbolt-Universität zu Berlin, 10117 Berlin, Germany; 2 Department of Internal Medicine II, Center for Complementary Medicine, Freiburg University Hospital, Faculty of Medicine, University of Freiburg, 79106 Freiburg, Germany; 3 Department of Internal and Integrative Medicine, Immanuel Hospital Berlin, 14109 Berlin, Germany

**Keywords:** plant-based diet, nutrient supply, cardiovascular risk, dietary intake, vegan, vegetarian, micronutrients, macronutrients

## Abstract

A plant-based diet (PBD) can provide numerous health benefits for patients with cardiovascular risk factors. However, an inadequately planned PBD also bear the potential for deficiencies in certain macro- and micronutrients. The present study analyzed nutrient profiles of individuals who adopted a PBD as part of the CardioVeg study. Participants with cardiovascular risk factors were randomly assigned to either a whole-food PBD intervention (*n* = 36; eight 90 min group meetings including two 120 min cooking sessions) or a control group asked to maintain an omnivorous diet (*n* = 34) for eight weeks. Food intake data were collected using three-day weighed food records and analyzed with NutriGuide software, including the German Nutrient Data Base (German: Bundeslebensmittelschlüssel). Nutrient intake was compared before and after eight weeks as well as between the groups. The results for both groups were then contrasted to the current dietary recommendations published by the societies for nutrition in Germany, Austria, and Switzerland. Moreover, anthropometric/laboratory data and ambulatory blood pressure monitoring were determined at baseline and after 8 weeks. Data of a subsample (*n* = 18 in the PBD group and *n* = 19 in the control group) were used for the present analyses of the dietary intake data. A PBD yielded several benefits including (but not limited to) a lower energy density, a lower intake of cholesterol and saturated fat, an increased consumption of fiber, and a lower intake of salt. Recommended intakes of most vitamins and minerals were generally met, except for vitamin B12 in the PBD group. A low intake of several other critical nutrients (vitamin D, iodine) was observed in both groups. Compared with the control group, PBD resulted in a significant decrease in body weight, body mass index, waist circumference, HbA1c, and fasting blood glucose after 8 weeks. Overall, it can be concluded that a PBD had a more favorable nutrient composition for cardiovascular health than the omnivorous dietary pattern of the control group.

## 1. Introduction

Plant-based diets (PBD) are becoming increasingly popular for their many health benefits, both in the prevention and treatment of disease. PBD have been shown to convey protective effects against obesity, diabetes, and other metabolic disorders [1,2,3]. In addition, there is mounting evidence that a plant-based diet is beneficial for heart health [4,5,6,7,8,9].

PBDs maximize the consumption of nutrient-dense plant foods while minimizing (or eliminating) processed foods, oils, and animal products [10,11]. Thus, PBD are abundant in vegetables, fruits, legumes, and other unprocessed plant products. Systematic reviews and meta-analyses have demonstrated that the intake of fruits and vegetables [12,13,14,15,16], legumes [17], dietary fiber [18], nuts [19], and unsaturated fatty acids [20] provide multiple health benefits and are associated with a reduced frequency of cardiovascular events. The consumption of animal products (including red and processed meats) on the other hand is associated with an increased cardiovascular risk [21].

Results from the Adventist cohort study showed that people who eat a PBD reduced their risk of developing hypertension by almost 75% [22]. Vegetarian diets were also associated with significantly lower medical care expenditure in patients with cardiovascular disease and were suggested as an effective strategy to alleviate the medical-economic burden in selected populations [23].

Although PBD may offer numerous health benefits, it is often claimed that inadequately planned and non-diversified PBDs bear the potential of macro- and micronutrient deficiencies [24,25]. According to the Deutsche Gesellschaft für Ernährung (DGE, German Nutrition Society), it is “difficult or impossible to achieve an adequate supply of some nutrients with a purely plant-based diet” [26]. Vitamin B12, among others, is the most critical nutrient [26]. Further potentially critical nutrients are protein, long-chain n-3 fatty acids, as well as other vitamins (riboflavin, vitamin D) and minerals (calcium, iron, iodine, zinc, and selenium) [26].

We conducted a randomized controlled trial (the “CardioVeg” study) to investigate the effects of a PBD on cardiovascular risk factors. The aim of this dietary intake data analysis was to evaluate the macro- and micronutrient intake before and after an eight-week PBD intervention in patients with cardiometabolic risk factors. The results were contrasted with the current dietary recommendations published by the Societies for Nutrition in Germany (DGE), Austria (Österreichische Gesellschaft für Ernährung, ÖGE) and Switzerland (Schweizerische Gesellschaft für Ernährung, SGE)—the so-called D-A-CH (D—Deutschland, Germany), A—Austria, CH—Confoederatio Helvetica, Switzerland) recommendations [27]. We hypothesized that a properly composed PBD diet could meet all D-A-CH recommendations for macro- and micronutrient (except for vitamin B12) and may even excel with a beneficial dietary composition for cardiovascular health.

## 2. Materials and Methods

The CardioVeg study was a randomized controlled trial that examined the effects of PBD on health outcomes in relation to cardiovascular risk factors. Participants with an increased cardiometabolic risk (see Table 1) were randomized to follow a PBD (plant-based group, PBG) or to continue an omnivorous diet (waiting list control group, CG).

The CardioVeg study had been approved by the ethics committee of the Charité-Universitätsmedizin Berlin (approval number: EA4/025/19). Written, informed consent was obtained from all participants. The study was registered at ClinicalTrials.gov (NCT03901183) prior to patient recruitment. The present analysis is limited to a subsample of participants in the CardioVeg study. Only individuals that submitted a complete and plausible dietary protocol were considered. Further clinical parameters will be reported elsewhere. The allocation was based on a computer-generated randomization protocol and was supervised by a certified biostatistician. Due to an obvious lifestyle intervention, the assignment could not be blinded for participants.

### 2.1. Dietary Intervention

Participants in the PBG were asked to follow an ad libitum whole-food PBD, consisting of vegetables, grains, legumes, and fruits. We instructed participants to avoid animal products to the greatest extent possible [11,28]. The term PBD is frequently used as an umbrella term comprising various dietary patterns: veganism (complete avoidance of animal products), pescetarianism (including seafood), ovo-vegetarianism (including eggs), lacto-vegetarianism (including dairy products), ovo-lacto-vegetarianism (including eggs and dairy products) [28,29,30]. In our study, participants were free to choose their dietary pattern from the above-mentioned selection. All participants received nutritional counseling to establish a healthy whole-food PBD. The sessions were held by certified dietitians and nutrition scientists within eight group sessions of 90 min over a total period of 8 weeks in Berlin. During the counseling sessions, the nutritionists illustrated a whole-food plant-based diet. The sessions were structured into themes such as healthy plant-based proteins, fats, complex carbohydrates, vitamins, etc. Food recipes were handed out and substitutes for animal foods were recommended. At the weekly meetings, progress was shared initially, and participants exchanged their experience with the nutritionist. The consultation included 2 cooking sessions (120 min each) focusing on practical suggestions to implement a well-balanced PBD. Due to the COVID-19 pandemic, external regulations forced us to conduct the nutrition course online after inclusion of half of the subjects. An 8-week intervention period represents a time frame that is considered acceptable, and not too long to commit to weekly counseling sessions, and at the same time long enough to develop healthy habits [31].

The waiting list CG was instructed to maintain their current omnivorous diet but was offered to participate in the nutritional counseling program after completion of the last study visit.

Participants received no remuneration.

### 2.2. Dietary Intake and Monitoring

Dietary intake was assessed using 3-day weighed food records (3 consecutive days, with 2 weekdays and one weekend day). Participants were instructed and given templates to accurately protocol food intake (portion sizes of various foods and beverages consumed). These records were logged by all participants at baseline and after 8 weeks at the same time of the study visits, when also laboratory and anthropometric measurements were assessed (see Section 2.3).

Dietary intake data were collected and digitalized by a nutrition scientist, using the Software NutriGuide 4.7 Plus (Nutri-Science GmbH, Hausach, Germany). NutriGuide performs its analysis based on the nutritional charts of the German Nutrient Data Base (German: Bundeslebensmittelschlüssel, BLS 3.02), containing about 14.800 food items split by their nutrients. Three-day average values for energy, carbohydrate, protein, fat, and micronutrient intake were calculated. Absolute values and percentage values in relation to the Daily Recommended Intake (DRI) of the D-A-CH were used for further analysis. The D-A-CH reference values for nutrient intake are published collaboratively by the Societies for Nutrition in Germany (DGE), Austria (ÖGE) and Switzerland (SGE) [27].

The daily recommended intake was individually adjusted to gender, age, and estimated to the physical activity level (PAL) of 1.6 indicating a sedentary lifestyle (with occasionally additional energy expenditure for walking and standing activity). The gender- and age-specific DRI for a PAL of 1.6 can be obtained from the Supplementary Materials, Table S1 [27].

### 2.3. Anthropometric/Laboratory Data and Blood Pressure

Anthropometric and laboratory data as well as ambulatory blood pressure monitoring (ABPM) were determined at baseline and after 8 weeks. Blood tests assessed blood sugar, insulin resistance (Homeostasis Model Assessment, HOMA-Index), hemoglobin A1c (HbA1c), triglycerides, low-density lipoprotein (LDL), and high-density lipoprotein (HDL) cholesterol levels. Blood samples were collected after a 10 h overnight fast from the antecubital vein into vacutainer tubes and analyzed using the Modular P analyzer (Roche, Mannheim, Germany). 

Trained staff measured participants’ weight and height, which was used to calculate body mass index (BMI). Abdominal obesity was determined by waist circumference, which was measured by the study nurse at midpoint between the last rib and the iliac crest. Twenty-four-hour ambulatory systolic and diastolic blood pressure were measured using a digital blood pressure monitor validated for clinical studies (Spacelabs 90217A). The monitoring software automatically removed incorrect measurements using build-in algorithms. 

### 2.4. Statistical Analysis

SPSS Version 27.0 and Microsoft Excel were used to complete all statistical analyses. A *p*-value of <0.05 was used to determine statistical significance.

- Data are presented as means ± standard deviations and 95% confidence interval
- For laboratory data the Shapiro–Wilk test was used to determine normality.
- When normality was confirmed, participants characteristics and biochemistry was analyzed with a two samples t-test to assess differences between groups.
- Dietary nutrient intake was compared within the groups with the related-samples Wilcoxon signed rank test.
- Treatment effect and *p*-value between groups was determined using the Mann–Whitney-U test, comparing the difference of nutrient intake (Δ = intake at baseline vs. intake after 8 weeks). The difference is depicted as mean and 95% confidence interval (CI).

## 3. Results

### 3.1. Randomization/Participants

**Figure 1:**
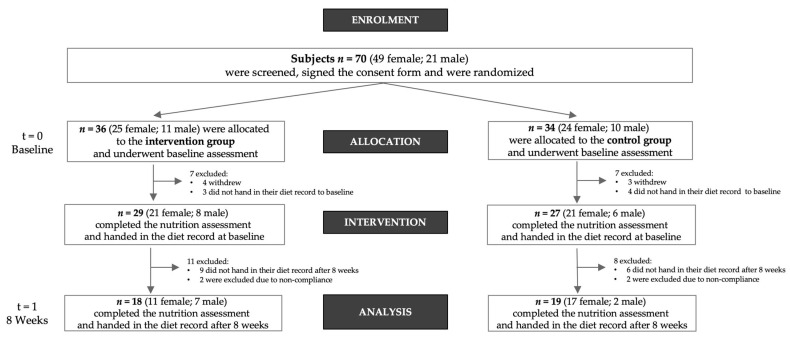
Flowchart of the study population.

Participants (*n* = 70) with increased cardiometabolic risk factors were randomized to follow a plant-based (*n* = 36) or to continue an omnivorous diet (*n* = 34). Patients were recruited between May 2019 and February 2021. From initially 70 participants recruited to complete the CardioVeg study, 7 participants withdrew. Twenty-two participants did not return their dietary records for nutritional analyses and were thus excluded from the present analysis. We removed four participants due to noncompliance with the study protocol. A total sample size of 37 (*n* = 18 in the PBG and *n* = 19 in the CG) was used for the present analyses of the dietary intake data. Figure 1 shows the participant inclusion flow chart for the present analysis.

The majority of participants of this subgroup were women: 61.1% in the PBG (*n* = 18) and 89.5% in the CG (*n* = 19). The mean age of participants in the PBG was 61.1 ± 7.0 years and 62.8 ± 7.0 years in the CG. 

### 3.2. Anthropometric/Laboratory Data and Blood Pressure

Table 2 shows anthropometric and laboratory data at baseline and after 8 weeks. The PBD resulted in a significant decrease in body weight after 8 weeks (mean difference [95% CI] = −3.5 kg [−5.3–−1.7]), BMI (−1.2 kg/m^2^ [−1.8–−0.6]), and waist circumference (−3.2 cm [−5.1–−1.3]), see Table 2. In addition to that, we observed a significant reduction in HbA1c (−1.7 mmol/mol [−2.8–−0.6]) and fasting blood glucose levels (−5.8 mg/dl [−9.1–−2.6]) in the PBG. Compared to the CG, all above values were significant (Table 2). HOMA Index, triglycerides, cholesterol (LDL, HDL) as well as ABPM were not significant between the groups, although PBG showed more favorable effects. 

### 3.3. Results of the Dietary Intake Data

All 37 participants of this subgroup analysis followed an omnivorous diet before the intervention. According to the food records, 19 participants of the CG remained their omnivorous diet for the course of the study. Participants of the PBG adjusted their diet as follows: Eleven participants adopted a strict vegan diet. Four participants adopted a lacto-vegetarian diet, two adopted a lacto-ovo-vegetarian and one participant switched to a pesco-vegetarian diet.

**Figure 2:**
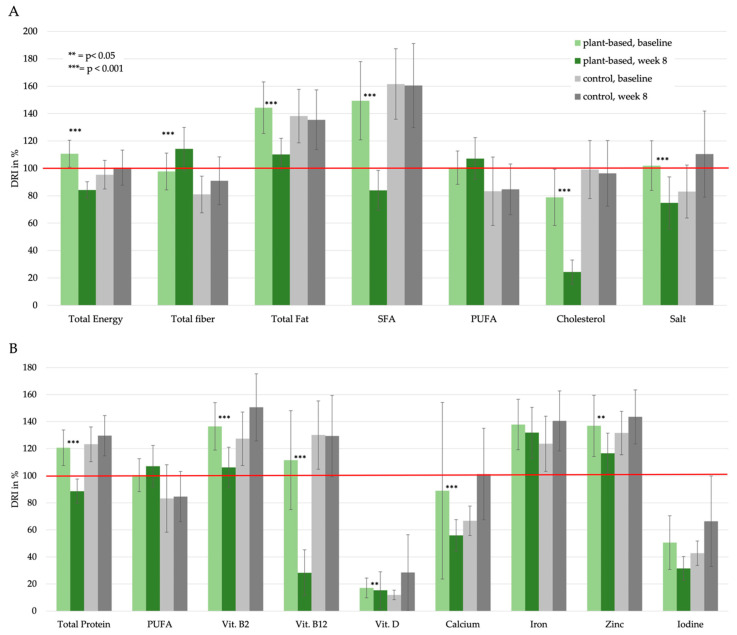
Nutrient intake in relation to the D-A-CH reference values: (**A**) potential beneficial nutrients in a PBD; (**B**) potential critical nutrients in a PBD. The error bars represent the 95% Confidence Interval of the average daily nutrient intake. *p*-value is based on the comparison of absolute values within the group and assessed by related-samples Wilcoxon signed rank test (absolute values are listed in Table S2).

Ultimately, we examined the effects of the plant-based intervention on diet quality. Mean daily intakes of the major nutrient components and the percentage of adequate nutrient intake (adjusted to gender, age and physical activity) in relation to the D-A-CH recommendations are shown in Table 3 and in detail in Table S2 in the Supplementary Materials. Figure 2 shows nutrient intakes in relation to D-A-CH reference values: Potentially beneficial nutrients in a PBD are shown in section A. Potentially critical nutrients in a PBD are plotted in section B. 

### 3.4. Macronutrient Intake

In terms of macronutrient intake, there were following significant between-group differences after 8 weeks: total daily intakes of energy, total protein, total fat, and cholesterol were significantly lower in the PBG (all *p* < 0.001 between the groups). The PBG consumed significantly less saturated fatty acids (SFA, *p* < 0.001) and less monounsaturated fatty acids (MUFA) (*p* = 0.001). Polyunsaturated fatty acid (PUFA) intake increased slightly, but the difference was not significant between the groups (*p* = 0.129). PBG participants consumed significantly more dietary fiber (*p* = 0.002) and Alpha-Linolenic Acid (ALA) (*p* = 0.013) than participants in the CG.

### 3.5. Micronutrient Intake/Vitamins

We observed a significant decrease in the intake of essential vitamins (vitamins B2, B3, B5, B6, B12, and vitamin D) within the PBG. Retinol equivalent, vitamin B1, biotin and folate were all slightly reduced but the decrease was not statistically significant within the PBG. Concerning vitamin C and vitamin E there was a modest but not significant increase within the PBG. Glancing at potential between-group differences, only vitamin B3, B6, and B12 differed significantly.

### 3.6. Micronutrient Intake/Minerals

Compared to the CG, intake of certain minerals significantly decreased in the PBG: Sodium intake decreased by more than 1.1 g in the intervention group (*p* < 0.001). Additionally lower chloride (*p* < 0.001), potassium (*p* = 0.021), zinc (*p* < 0.001), sulfur (*p* < 0.001), phosphorus (*p* < 0.001), calcium (*p* < 0.001), and iodine (*p* < 0.001) were present in the PBG compared to the CG. 

## 4. Discussion

The primary aim of the present dietary data analysis was to contrast the nutritional quality of a PBD to an omnivorous diet. Moreover, we sought to examine whether a properly composed whole-food PBD could meet all D-A-CH recommendations. We put a major focus on nutrients of potential public health concern [26,32]. Our data suggest that the PBD had various beneficial components including but not limited to a lower energy density, a lower intake of cholesterol and saturated fat, an increased consumption of dietary fiber. and a lower intake of salt. It is worth mentioning that most participants voluntarily chose a purely “vegan diet”.

### 4.1. Potential Beneficial Nutrient Intake in a PBD

#### 4.1.1. Energy Intake 

Excess weight, as shown by a higher BMI or waist circumference, is one of the strongest risk factors for cardiovascular disease [33]. Plant-based foods are characterized by lower energy density and a higher nutrient density. Thus, they tend to promote weight loss [28]. Consistent with our results, the PBG consumed significantly fewer calories compared to the CG although neither group had any quantity restrictions. It is conceivable that the reduced energy density contributed to weight loss in the PBG [34].

#### 4.1.2. Dietary Fiber Intake

Other nutrient-related benefits of the PBD intervention included a high intake of dietary fiber. Dietary fiber is a component of plant foods that cannot be broken down by enzymes in the human gastrointestinal tract. Its consumption reduces the risk of obesity in adults, as well as the risk of hypertension and coronary heart disease [35,36]. By lowering total and LDL cholesterol concentrations, dietary fiber also diminishes the risk of dyslipidemia [37,38,39,40]. As a guideline, D-A-CH recommends a dietary fiber intake of at least 30 g/day. Participants in the PBG achieved this recommendation, while the CG failed.

#### 4.1.3. Saturated Fatty Acids (SFA) and Cholesterol Intake

Participants allocated to the PBG consumed less SFA and less MUFA while their intake of PUFA increased slightly but not significantly. SFA content is particularly low in a PBD [41,42], which has been linked to coronary heart disease prevention by improving lipid profiles and lowering blood pressure [43,44,45]. Notably, the between-group differences were also significant: While CG substantially exceeded the recommended daily intake of SFA, the PBG was able to successfully reduce its consumption below the limit.

Throughout the study the intake of PUFAs increased slightly in the PBG but not significantly. There was no insufficient intake before and after the intervention in the PBG. Differently, the intake of PUFAs in the CG was already low at the beginning of the study and remained low during the 8 weeks of the study. Plant foods contain just small amounts of MUFA and PUFA, mainly α-linolenic acid (ALA). ALA is a short-chain n-3 PUFA that occurs in plant derived sources such as vegetable oils, walnuts, rapeseed, linseed, and hemp. ALA can be converted to a limited extent to essential omega-3 fatty acids (eicosapentaenoic acid and docosahexaenoic acid) that are known to be cardioprotective [29,44,46,47].

A high intake of saturated fat has been shown to adversely affect serum LDL concentrations [48]. Moreover, several studies suggested an association between dietary cholesterol and serum cholesterol [49,50]. While some international dietary associations have removed the target values for dietary cholesterol, D-A-CH maintains its recommendation and still advises limiting cholesterol intake to about 300 mg per day. Both study groups did not exceed this recommendation. However, it is evident that subjects in the PBG group consumed significantly less dietary cholesterol than subjects in the CG group and were able to reduce this consumption during the intervention. 

Despite a close relationship between SFA, cholesterol intake and blood lipid levels, our analysis did not show significant results regarding lipid panels. One potential reason is the short intervention duration. Non-statistical differences may also be a result of under-powering (see Section 5).

#### 4.1.4. Salt Intake

Sodium and chloride are essential for various metabolic pathways and fluid regulation, however, a high consumption of salt is a major cause of hypertension and an independent risk factor for coronary heart disease and stroke [51]. There is consistent evidence that a moderate reduction in salt intake (i.e., a reduction of 3 to 5 g) can lead to a decrease in blood pressure [52,53]. Although the physiological requirement is only 2 to 3 g per day, the D-A-CH recommends a maximum of 5 g per day. The PBG group managed to significantly lower their salt intake from 6.5 ± 2.0 g to 3.7 ± 2.1 g, while the CG did not show any decrease.

### 4.2. Potential Critical Nutrients in a PBD

Our data suggest that participants allocated to the PBG consumed adequate amounts of macronutrients and essential vitamins and met the D-A-CH recommendations in most cases. 

#### 4.2.1. Protein Intake

The adequacy of protein intake in PBDs is controversial. Proteins are required for the structure, function, and regulation of the body’s cells, tissues, and organs, and each protein has unique functions. Although protein-rich plant foods such as traditional legumes, nuts, and seeds may be sufficient to achieve complete protein intake in adults following a PBD, our dietary data analysis showed otherwise. The PBG consumed only 89% [95% CI: 89;98] protein, which is 11% less than recommended by D-A-CH.

#### 4.2.2. Critical Micronutrients

According to the German Nutrition Society, a strict PBD does not provide an adequate supply of some nutrients or provides them only with difficulty. Potentially critical nutrients in a vegan diet include vitamins (vitamin B12, riboflavin/vitamin B2 and vitamin D) as well as certain minerals (calcium, iron, iodine, zinc, selenium) [26]. The most critical nutrient is certainly vitamin B12 [26]. As expected, vitamin B12 intake in the PBG decreased significantly below the DRI. Since vitamin B12 is an important component of various metabolic pathways, it is strongly recommended to supplement this essential nutrient when adopting a PBD.

In our analysis riboflavin, also known as vitamin B2, also declined slightly in the PBG, yet it remained above the recommended daily intake. As for vitamin D and pantothenic acid (vitamin B5), both groups showed inadequate intakes, suggesting that these nutrients are not only critical for vegans, but nutrients of public health concern. 

Pantothenic acid is a water-soluble vitamin and a precursor for the synthesis of coenzyme A. In fact, coenzyme A is essential for many biochemical reactions that maintain life [54].

Vitamin D is essential for maintaining bone mineralization by regulating calcium and phosphorus homeostasis. However, a deficiency has not only negative effects on the human skeletal system but also facilitates the development and progression of numerous common diseases, including cardiovascular disease, diabetes, autoimmune diseases, and cancer [55].

The intake of the trace element iodine was also insufficient in both groups. Iodine is an essential component of thyroid hormones, which are needed throughout life for normal growth, neurological development and metabolism. Insufficient iodine intake impairs the production of thyroid hormones and leads to a condition called hypothyroidism. This leads to a range of health impairments of varying severity [56].

Calcium intakes decreased in the PBG and did not meet the D-A-CH guideline. As a major component of bones and teeth, calcium also plays an important role as a second messenger in cell signaling pathways [57].

Other critical nutrients such as iron, and zinc decreased in PBG, but levels were still above recommended values. 

## 5. Limitations

The present study has several strengths and limitations that warrant further discussion. We conducted most of the study under pandemic conditions—external regulations and lockdowns forced us to switch from face-to-face training to online sessions. Despite these difficulties, we managed to recruit a total of 70 people. The main limitation of this subsample analysis is that not all participants provided plausible and complete food records. Therefore, the current analysis is limited to 37 participants. Our study may thus be underpowered and unable to detect smaller group differences.

Adopting a PBD may be difficult in the first weeks and requires external support. It is conceivable that online education sessions are less effective and do not allow for the same personal interaction that is possible during in-person events. Whether this affected adherence in the PBG, however, remains a subject to speculations. 

Another limitation of this study results from the dietary protocols: The direct form of a dietary survey by keeping protocols causes a higher awareness among the participants. This may lead to a more conscious perception of their own diet. Foods that are assumed to be positively evaluated by the investigator (e.g., vegetables, fruits) are usually overestimated in quantity or even consumed more frequently during the protocol days. In contrast, other foods that are considered undesirable (e.g., sweets, alcoholic beverages) tend to be underestimated or consumed less. This effect, which is desirable in nutrition education, is a potential source of error in the analysis of our nutrition data.

Although three-day weighed food records are the gold standard in nutritional monitoring, they are also susceptible to various bias, including reporting bias. More solid results on nutrient absorption and acquired deficiencies can be obtained by blood analysis. In our study, we focused on dietary intake and omitted blood tests regarding micro-/macronutrients; however, we would recommend and perform them in future studies. Concerning our study, it would be particularly interesting to determine in the blood whether the critical nutrients were too low in the intake but possibly still sufficiently present in the organism. Furthermore, these parameters could be complemented by microbiome and multi-omics data, since our microbiota produces vitamins, among other substances, and thus contributes to a healthy diet [58].

## 6. Conclusions

The present analysis of dietary intake showed that the nutrient composition of participants in the whole-food PBG was more favorable for cardiovascular health compared with participants to the omnivorous CG. Beneficial features of the PBD included a lower energy density, a lower intake of SFA and cholesterol, an increased consumption of dietary fiber, and a lower intake of salt. The recommended intake for most vitamins and minerals were met. As expected, participants in the PBG did not meet the recommendations for vitamin B12, and supplementation may thus be warranted. A low intake of several critical nutrients (vitamin D, pantothenic acid, and iodine) was observed in both groups, suggesting that these are nutrients of public health concern. Targeted supplementation with the previously mentioned micronutrients could improve the nutritional quality of the PBD and prevent the development of nutritional deficiencies. Overall, however, the benefits and the preventive effect that PBD offers for heart health are so valuable that we recommend PBD as adjunct therapy to the patient’s medication and usual diet.

## Figures and Tables

**Table 1:** Inclusion and exclusion criteria for the CardioVeg study.

Inclusion Criteria	Exclusion Criteria
Men and women aged 25 to 75 and diagnosed with: hypertension (from >140 mmHg systolic and/or >90 mmHg diastolic), central obesity (waist circumference > 94 cm for men, >80 cm for women), A non-vegetarian diet in the past 6 months (at least 4× meat and/or meat products per week, at least 5× dairy products per week) No fasting, no specific diet or change of diet in the last 2 months Weight stable over the last two months (±3 kg) Medication unchanged for at least one month	a poor general condition diagnosed coronary heart disease diabetes mellitus type I cerebrovascular disease severe mental illness severe acute or chronic comorbidity pregnancy and lactation or planned pregnancy in the next 6 months eating disorder alcohol consumption more than 2 beers 0.5l or 2 wines 0.2l per day no alcohol abstinence 48 h before blood samples possible over 5 cigarettes/day medication that affect weight antibiotics within the last 6 months major surgery <6 months prior to randomization BMI over 40 kg/m^2^ existing vegetarian or plant-based diet bariatric surgery simultaneous participation in another clinical trial participation in a clinical trial within the last 3 months prior to inclusion in the study lack of consent to participate in the study

**Table 2:** Characteristics of the study population before and after 8 weeks.

	Plant-Based Group (*n* = 18)			Control Group (*n* = 19)			
	Baseline	Week 8	Δ [95% CI]	Baseline	Week 8	Δ [95% CI]	*p*-Value *
**Anthropometrics**							
Weight [kg]	93.0 ± 16.6	89.5 ± 15.5	−3.5 [−5.3–−1.7]	80.7 ± 11.9	80.4 ± 12.1	−0.3 [−1.1–0.5]	0.002
Body mass index [kg/m^2^]	31.7 ± 4.6	30.5 ± 4.1	−1.2 [−1.8–−0.6]	29.5 ± 4.5	29.4 ± 4.8	−0.1 [−0.4–0.3]	0.002
Waist circumference [cm]	109.9 ± 11.1	106.7 ± 9.4	−3.2 [−5.1–−1.3]	101.2 ± 7.0	101.5 ± 7.1	0.2 [−0.9–1.4]	0.004
**Laboratory data**							
Fasting blood glucose [mg/dl]	99.7 ± 15.5	93.9 ± 12.9	−5.8 [−9.1–−2.6]	93.1 ± 16.2	92.5 ± 15.1	−0.6 [−3.9–2.8]	0.042
HbA1c [mmol/mol]	40.1 ± 6.5	38.4 ± 5.4	−1.7 [−2.8–−0.6]	36.5 ± 3.1	36.8 ± 3.8	0.3 [−0.5–1.2]	0.009
HOMA Index	3.7 ± 2.7	2.9 ± 1.8	−0.8 [−1.4–−0.1]	2.6 ± 1.9	2.4 ± 1.7	−0.2 [−0.6–0.3]	0.170
Triglycerides [mg/dl]	112.1 ± 36.5	126.6 ± 48.5	14.6 [−2.5–31.6]	120.1 ± 58.1	135.6 ± 76.7	15.6 [−1.0–32.2]	0.936
Cholesterol [mg/dl]	214.1 ± 26.8	198.7 ± 28.1	−15.4 [−27.4–−3.5]	227.4 ± 46.1	223.9 ± 53.7	−3.5 [−15.5–8.4]	0.191
LDL [mg/dl]	137.3 ± 26.0	125.0 ± 27.6	−12.3 [−23.9–−0.7]	147.4 ± 44.8	147.0 ± 52.7	−0.4 [−11.6–10.8]	0.171
HDL [mg/dl]	63.6 ± 15.9	56.3 ± 13.4	−7.4 [−10.1–−4.7]	66.3 ± 21.1	62.4 ± 20.2	−3.9 [−6.9–−0.9]	0.117
**Ambulatory blood pressure monitoring**							
ABPM SBP [mm Hg]	135.9 ± 11.0	130.3 ± 14.7	−5.6 [−10.6–−0.5]	130.6 ± 13.3	131.9 ± 13.1	1.3 [−3.5–6.1]	0.088
ABPM DBP [mm Hg]	83.3 ± 8.8	80.1 ± 9.3	−3.2 [−6.2–−0.3]	76.9 ± 5.8	78.0 ± 6.8	1.1 [−1.8–3.9]	0.069

Data is presented as mean ± SD; the difference is depicted as mean and 95% Confidence Interval; * *p*-value between groups was determined using a two samples *t*-test.

**Table 3:** Absolute and relative daily nutrient intake before and after 8 weeks and comparison between the groups (extract from Table S2 in the Supplementary Materials).

	Plant-Based Group (*n* = 18)				Control Group (*n* = 19)				
	Baseline		Week 8		Baseline		Week 8		*p*-Value ^b^
	Intake ^a^	% of DRI ^c^	Intake	% of DRI	Intake	% of DRI	Intake	% of DRI	
**Macronutrients**									
Energy [kcal]	2392.2 ± 382.6	111 [101;121]	1798.1 ± 315.1	84 [78;90]	1955.4 ± 452.0	95 [85;106]	1955.1 ± 477.1	101 [88;113]	<0.001
Total carbohydrates [g]	217.2 ± 58.9	74 [65;82]	189.7 ± 41.6	69 [62;75]	178.8 ± 52.7	64 [54;73]	187.9 ± 47.2	74 [60;89]	0.078
Total dietary fiber [g]	24.2 ± 8.7	98 [84;111]	31.3 ± 8.6	114 [98;130]	24.9 ± 8.3	81 [68;94]	24.5 ± 8.1	91 [74;108]	0.002
Total Protein [g]	90.3 ± 15.0	121 [108;134]	56.2 ± 10.1	89 [80;98]	74.4 ± 15.5	123 [110;136]	75.8 ± 18.4	123 [110;136]	<0.001
Total Fat [g]	112.6 ± 22.3	144 ± 41 [125;163]	78.1 ± 18.3	110 [98;122]	91.3 ± 29.5	138 [119;158]	87.5 ± 30.8	135 [114;157]	0.005
SFA [g]	45.3 ± 12.7	149 [121;178]	21.3 ± 9.0	84 [70;98]	35.6 ± 12.8	162 [136;187]	34.7 ± 14.7	161 [130;191]	<0.001
MUFA [g]	41.1 ± 9.1	157 [134;180]	28.3 ± 8.2	123 [105;141]	31.2 ± 10.5	143 [122;164]	29.5 ± 10.6	136 [113;158]	0.001
PUFA [g]	18.3 ± 5.3	101 [88;113]	23.7 ± 8.4	107 [92;122]	18.4 ± 12.1	83 [58;108]	17.6 ± 8.4	85 [66;103]	0.129
LA [g]	14.4 ± 5.2	290 [246;333]	19.1 ± 7.4	300 [244;357]	14.0 ± 9.4	253 [177;330]	14.1 ± 7.6	250 [181;318]	0.191
ALA [g]	2.5 ± 1.8	351 [280;421]	4.1 ± 3.3	375 [231;519]	3.2 ± 3.8	293 [133;453]	2.3 ± 2.1	224 [139;309]	0.013
Cholesterol [mg]	383.7 ± 133.1	79 [58;99]	76.7 ± 58.8	24 [16;33]	301.9 ± 142.6	99 [78;120]	294.7 ± 163.8	96 [72;120]	<0.001
Salt [g]	6.5 ± 2.0	102 [84;120]	3.7 ± 2.1	75 [56;94]	4.3 ± 2.0	83 [64;102]	5.0 ± 1.8	110 [79;142]	<0.001
**Vitamins**									
Retinol equivalent [µg]	1660.6 ± 865.3	169 [119;219]	1230.4 ± 771.3	140 [95;184]	1575.9 ± 8401	173 [141;206]	1578.3 ± 632.6	193 [155;230]	0.202
Vitamine B1 [mg]	1.4 ± 0.3	133 [118;148]	1.4 ± 0.4	128 [110;145]	1.2 ± 0.3	111 [98;125]	1.3 ± 0.4	130 [110;150]	0.136
Vitamine B2 [mg]	1.7 ± 0.4	136 [119;154]	1.1 ± 0.3	106 [92;121]	1.4 ± 0.4	127 [108;147]	1.5 ± 0.5	151 [126;175]	0.242
Vitamine B3, Niacin equivalent [mg]	38.3 ± 8.1	268 [229;307]	24.7 ± 5.5	195 [166;224]	31.1 ± 7.8	269 [235;304]	30.1 ± 8.2	258 [224;292]	<0.001
Vitamine B5 [mg]	5.0 ± 1.2	80 [71;90]	4.0 ± 1.6	69 [56;81]	4.5 ± 1.2	71 [62;81]	4.7 ± 1.7	85 [67;103]	0.068
Vitamine B6 [mg]	1.8 ± 0.4	132 [117;146]	1.5 ± 0.4	117 [102;132]	1.6 ± 0.3	127 [113;141]	1.6 ± 0.4	131 [116;145]	0.005
Vitamine B7, Biotin [µg]	52.3 ± 16.3	118 [102;135]	48.5 ± 14.8	109 [93;125]	46.3 ± 11.7	97 [84;110]	49.5 ± 15.2	116 [98;134]	0.288
Vitamine B9, Folate [µg]	350.9 ± 109.1	115 [98;131]	310.9 ± 70.6	109 [99;118]	291.6 ± 94.3	94 [79;109]	292.1 ± 75.9	103 [88;118]	0.236
Vitamine B12 [µg]	5.7 ± 2.4	112 [75;148]	1.0 ± 1.2	28 [11;45]	3.9 ± 1.7	130 [105;155]	4.0 ± 2.0	129 [99;159]	<0.001
Vitamine C [mg]	125.5 ± 54.3	144 [120;167]	144.1 ± 84.6	160 [121;200]	157.1 ± 80.1	175 [135;214]	126.4 ± 47.3	127 [104;150]	0.121
Vitamine D [µg]	3.8 ± 3.2	17 [10;24]	1.7 ± 1.5	15 [2;29]	3.9 ± 6.3	12 [8;15]	2.7 ± 1.5	28 [−1;58]	0.136
Vitamine E [mg]	16.8 ± 6.0	155 [134;177]	19.8 ± 5.4	163 [141;184]	16.1 ± 7.7	142 [111;172]	18.0 ± 8.0	152 [119;185]	0.574
Vitamine K [µg]	195.6 ± 193.3	254 [143;366]	152.2 ± 131.3	214 [117;310]	161.4 ± 108.5	246 [172;320]	190.2 ± 152.7	269 [162;376]	0.316
**Minerals**									
Sodium [mg]	2753.5 ± 822.2	109 [90;128]	1620.7 ± 870.7	81 [60;101]	1861.1 ± 863.5	90 [69;110]	2147.6 ± 820.4	119 [88;149]	<0.001
Chloride [mg]	4170.7 ± 1222.8	113 [94;132]	2563.5 ± 1298.8	86 [66;106]	2814.6 ± 1189.6	91 [72;110]	3291.0 ± 1150.5	117 [93;141]	<0.001
Potassium [mg]	3402.8 ± 651.3	160 [145;175]	2970.9 ± 655.5	144 [126;162]	3001.4 ± 627.3	144 [126;161]	3046.4 ± 581.7	149 [134;164]	0.021
Magnesium [mg]	498.1 ± 401.7	145 [83;208]	428.3 ± 107.4	131 [111;152]	348.4 ± 117.6	110 [91;129]	362.4 ± 99.6	127 [108;145]	0.715
Zinc [mg]	11.9 ± 2.6	137 [114;160]	8.9 ± 2.2	117 [102;131]	9.6 ± 2.5	132 [116;148]	10.6 ± 3.6	143 [123;164]	0.001
Copper [µg]	2260.1 ± 720.2	181 [154;207]	2372.7 ± 603.8	180 [152;208]	1896.4 ± 658.7	146 [119;173]	1931.5 ± 613.2	163 [136;189]	0.738
Phosphorus [mg]	1453.4 ± 258.4	187 [170;204]	1127.2 ± 303.5	156 [133;179]	1210.0 ± 246.9	165 [146;185]	1289.6 ± 331.4	187 [166;209]	<0.001
Fluoride [µg]	2004.3 ± 4179.9	45 [−17;108]	953.0 ± 501.6	31 [22;41]	789.0 ± 377.2	24 [18;30]	872.4 ± 435.6	58 [−1;116]	0.136
Calcium [µg]	1174.5 ± 1412.9	89 [24;154]	551.4 ± 188.7	56 [44;68]	708.4 ± 203.7	67 [56;78]	849.8 ± 260.5	101 [68;135]	<0.001
Iron [mg]	14.3 ± 3.8	138 [119;157]	13.8 ± 3.7	132 [113;151]	13.5 ± 4.0	124 [103;144]	13.6 ± 4.1	141 [118;163]	0.727
Iodine [µg]	121.9 ± 77.2	51 [31;71]	54.7 ± 25.5	32 [23;40]	91.9 ± 55.1	43 [34;52]	92.3 ± 28.0	66 [33;100]	<0.001
Manganese [µg]	5402.7 ± 2411.8	194 [162;226]	8038.3 ± 3563.5	243 [200;287]	5630.7 ± 2979.4	155 [114;196]	6324.4 ± 3642.3	218 [142;293]	0.019

Data results from three-day weighed food records analyzed with NutriGuide software, including the German Nutrient Data Base (German: Bundeslebensmittelschlüssel). ^a^ Nutrient intake is presented as mean ± SD and compared within the groups with the Wilcoxon signed rank test for paired samples. ^b^ Treatment effect and *p*-value between groups was determined using the Mann–Whitney U test, comparing the delta of the nutrient intake (=intake at baseline vs. intake after 8 weeks). ^c^ The adequate nutrient supply is depicted as mean [95% confidence interval]. It was calculated as a percentage of the daily recommended intake (DRI) and adjusted to gender and age and under the assumption of moderate movement (Physical Activity Level, PAL 1,6). D-A-CH Reference values are defined by the German (D), Austrian (A), and Swiss (CH) nutrition societies.

## Data Availability

Data from the study are available upon reasonable request.

## Supplementary Materials

The following supporting information can be downloaded at https://www.mdpi.com/article/10.3390/nu14214597/s1, Table S1. D-A-CH reference values: Gender- and age-specific DRI for a PAL of 1.6; Table S2. Absolute and relative daily nutrient intake before and after the intervention and comparison between the groups.

## Abbreviations

ALA	α-Linolenic Acid
ABPM	Ambulatory blood pressure monitoring
BMI	Body Mass Index
BLS	German Nutrient Data Base; german: Bundeslebensmittelschlüssel
CI	Confidence Interval
CG	Control Group
CVD	Cardiovascular Disease
D-A-CH	Association of nutritional societies of Germany (D), Austria (A) and Switzerland (CH)
DBP	24 h Diastolic Blood Pressure
DGE	Society for Nutrition in Germany (Deutsche Gesellschaft für Ernährung)
DRI	Daily Recommended Intake
HbA1c	Hemoglobin A1c
HDL	High Density Lipoprotein
HOMA	Homeostasis Model Assessment
LDL	Low Density Lipoprotein
MUFA	Monounsaturated Fatty Acids
ÖGE	Society for Nutrition in Austria
PAL	Physical Activity Level
PBD	Plant-Based Diet
PBG	Plant-Based Group
PUFA	Polyunsaturated atty Acids
SBP	24 h Systolic Blood Pressure
SD	Standard Deviation
SFA	Saturated Fatty Acids
SGE	Society for Nutrition in Switzerland
