# Supplementary material for: Does a Plant-Based Diet Stand Out for Its Favorable Composition for Heart Health? Dietary Intake Data from a Randomized Controlled Trial
Source: Nutrients. 2022 Nov 1;14(21):4597. doi: 10.3390/nu14214597 (PMC9656677; doi:10.3390/nu14214597)
Supplement: Supplementary file 1 [file nutrients-14-04597-s001.zip › nutrients-1969441-supplementary.pdf]

Supplementary Materials

Does a plant-based diet stand out for its favorable composition for heart health? Dietary intake data from a randomized-controlled trial

Table S1. D-A-CH reference values: Gender- and age-specific DRI for a PAL of 1.6

Nutrient	Unit	Women age 25-51	Women age 51-65	Women age 65<	Men age 25-51	Men age 51-65	Men age 65<
**Macronutrients**							
Total Energy	kcal	2100	2000	1900	2700	2500	2500
Total carbohydrates	g	287.5	273	260.1	369.6	342.3	342.3
Total dietary fiber	g	30	30	30	30	30	30
Total Protein	g	62.5	59.5	56.5	80.4	74,4	74.4
Total Fat	g	67.7	64.5	61.3	87.1	80.6	80.6
SFA	g	22.6	21.5	20.4	29	26.9	26.9
MUFA	g	22.6	21.5	20.4	29	26.9	26.9
PUFA	g	22.6	21.5	20.4	29	26.9	26.9
Linoleic Acid	g	5.6	5.7	5.1	7.3	6.7	6.7
Alpha-Linoleic Acid	g	1.1	1.1	1	1.5	1.3	1.3
Cholesterol	mg	300	300	300	300	300	300
Salt	g	5	5	5	5	5	5
**Vitamins**							
Retinol equivalent	µg	800	800	800	1000	1000	1000
Vitamine B1	mg	1	1	1	1.2	1.2	1.1
Vitamine B2	mg	1.1	1	1	1.4	1.3	1.3
Vitamine B3 (Niacin equivalent)	µg	12000	11000	11000	15000	15000	14000
Vitamine B5 (Pantothenic acid)	mg	6	6	6	6	6	6
Vitamine B6	mg	1.2	1,2	1.2	1.5	1.5	1.4
Vitamine B7 Biotin	µg	45	45	45	45	45	45
Vitamine B9 Folate	µg	300	300	300	300	300	300
Vitamine B12	µg	3	3	3	3	3	3
Vitamine C	mg	95	95	95	110	110	110
Vitamine D	µg	20	20	20	20	20	20
Vitamine E.	mg	12	12	11	14	13	12
Vitamine K	µg	60	65	65	70	80	80
**Minerals**							
Sodium	mg	2000	2000	2000	2000	2000	2000
Chloride	mg	3000	3000	3000	3000	3000	3000
Potassium	mg	2000	2000	2000	2000	2000	2000
Magnesium	mg	300	300	300	350	350	350
Zinc	mg	7	7	7	10	10	10
Copper	µg	1250	1250	1250	1250	1250	1250
Phosphorus	mg	700	700	700	700	700	700
Fluoride	µg	3100	3100	3100	3800	3800	3800
Calcium	mg	1000	1000	1000	1000	1000	1000
Iron	mg	15	10	10	10	10	10
Iodine	µg	200	180	180	200	180	180
Mangangese	µg	3500	3500	3500	3500	3500	3500

Table S2. Absolute and relative daily nutrient intake before and after the intervention and comparison between the groups

	Plant-Based (n=18)						Control (n=19)							
	Baseline		Week 8		Δ		Baseline		Week 8		Δ		Treat-ment effect	p-value[b]
	Intake[a]	% of DRI[c]	Intake	% of DRI	Intake	p-value[a]	Intake	% of DRI	Intake	% of DRI	Intake	p-value[a]		
**Macronutrients**														
Energy [kcal]	2392.2 ± 382.6	111 [101;121]	1798.1 ± 315.1	84 [78;90]	-594.1 ± 374.9	<0.001	1955.4 ± 452.0	95 [85;106]	1955.1 ± 477.1	101 [88;113]	-0.3 ± 357.0	0.777	0.66	<0.001
Total carbohydrates [g]	217.2 ± 58.9	74 [65;82]	189.7 ± 41.6	69 [62;75]	-27.5 ± 42.4	0.043	178.8 ± 52.7	64 [54;73]	187.9 ± 47.2	74 [60;89]	9.2 ± 58.9	0.616	0.29	0.078
Total dietary fiber [g]	24.2 ± 8.7	98 [84;111]	31.3 ± 8.6	114 [98;130]	7.1 ± 6.5	<0.001	24.9 ± 8.3	81 [68;94]	24.5 ± 8.1	91 [74;108]	-0.4 ± 8.0	0.777	0.50	0.002
Insoluble fiber [g]	15.9 ± 5.9	---	20.2 ± 5.2	---	4.4 ± 4.7	0.003	15.9 ± 4.8	---	15.2 ± 5.2	---	-0.6 ± 4.2	0.557	0.48	0.003
Soluble fiber [g]	7.1 ± 2.3	---	9.2 ± 2.9	---	2.1 ± 2.3	0.001	7.5 ± 2.1	---	7.6 ± 2.7	---	0.1 ± 2.8	0.679	0.39	0.016
Total protein [g]	90.3 ± 15.0	121 [108;134]	56.2 ± 10.1	89 [80;98]	-34.1 ± 13.3	<0.001	74.4 ± 15.5	123 [110;136]	75.8 ± 18.4	123 [110;136]	1.4 ± 17.4	0.679	0.78	<0.001
Essential AA [g]	43.9 ± 7.9	---	24.9 ± 5.9	---	-19.0 ± 6.6	<0.001	36.0 ± 8.1	---	36.1 ± 9.2	---	0.1 ± 8.5	0.983	0.80	<0.001
Non-essential AA [g]	44.2 ± 7.9	---	28.4 ± 6.0	---	-15.8 ± 6.3	<0.001	36.2 ± 7.3	---	36.5 ± 8.6	---	0.35 ± 8.1	0.948	0.78	<0.001
Total Fat [g]	112.6 ± 22.3	144±41 [125;163]	78.1 ± 18.3	110 [98;122]	-34.5 ± 31.0	0.001	91.3 ± 29.5	138 [119;158]	87.5 ± 30.8	135 [114;157]	-3.8 ± 26.9	0.286	0.46	0.005
SFA [g]	45.3 ± 12.7	149 [121;178]	21.3 ± 9.0	84 [70;98]	-24.1 ± 14.3	0.001	35.6 ± 12.8	162 [136;187]	34.7 ± 14.7	161 [130;191]	-0.9 ± 9.7	0.446	0.72	<0.001
MUFA [g]	41.1 ± 9.1	157 [134;180]	28.3 ± 8.2	123 [105;141]	-12.8 ± 12.4	0.003	31.2 ± 10.5	143 [122;164]	29.5 ± 10.6	136 [113;158]	-1.8 ± 9.8	0.267	0.55	0.001
PUFA [g]	18.3 ± 5.3	101 [88;113]	23.7 ± 8.4	107 [92;122]	5.4 ± 11.5	0.078	18.4 ± 12.1	83 [58;108]	17.6 ± 8.4	85 [66;103]	-0.7 ± 13.5	0.983	0.25	0.129
LA [g]	14.4 ± 5.2	290 [246;333]	19.1 ± 7.4	300 [244;357]	4.7 ± 10.3	0.071	14.0 ± 9.4	253 [177;330]	14.1 ± 7.6	250 [181;318]	0.2 ± 11.7	0.711	0.21	0.191
ALA [g]	2.5 ± 1.8	351 [280;421]	4.1 ± 3.3	375 [231;519]	1.6 ± 2.6	0.028	3.2 ± 3.8	293 [133;453]	2.3 ± 2.1	224 [139;309]	-1.0 ± 2.6	0.151	0.41	0.013
Cholesterol [mg]	383.7 ± 133.1	79 [58;99]	76.7 ± 58.8	24 [16;33]	-307.0 ± 144.8	<0.001	301.9 ± 142.6	99 [78;120]	294.7 ± 163.8	96 [72;120]	-7.2 ± 160.2	0.586	0.74	<0.001
Salt [g]	6.5 ± 2.0	102 [84;120]	3.7 ± 2.1	75 [56;94]	-2.8 ± 2.8	0.003	4.3 ± 2.0	83 [64;102]	5.0 ± 1.8	110 [79;142]	0.7 ± 2.0	0.286	0.60	<0.001
**Vitamins**														
Retinol equivalent [µg]	1660.6 ± 865.3	169 [119;219]	1230.4 ± 771.3	140 [95;184]	-430.1 ± 1275.4	0.184	1575.9 ± 8401	173 [141;206]	1578.3 ± 632.6	193 [155;230]	2.4 ± 1034.3	0.845	0.21	0.202
Vitamine B1 [mg]	1.4 ± 0.3	133 [118;148]	1.4 ± 0.4	128 [110;145]	-0.1 ± 0.5	0.632	1.2 ± 0.3	111 [98;125]	1.3 ± 0.4	130 [110;150]	0.1 ± 0.3	0.107	0.24	0.136
Vitamine B2 [mg]	1.7 ± 0.4	136 [119;154]	1.1 ± 0.3	106 [92;121]	-0.5 ± 0.4	<0.001	1.4 ± 0.4	127 [108;147]	1.5 ± 0.5	151 [126;175]	0.1 ± 0.4	0.157	0.19	0.242
Vitamine B3, Niacin equivalent [mg]	38.3 ± 8.1	268 [229;307]	24.7 ± 5.5	195 [166;224]	-13.6 ± 7.3	<0.001	31.1 ± 7.8	269 [235;304]	30.1 ± 8.2	258 [224;292]	-1.0 ± 7.6	0.528	0.67	<0.001
Vitamine B5 [mg]	5.0 ± 1.2	80 [71;90]	4.0 ± 1.6	69 [56;81]	-1.1 ± 1.6	0.012	4.5 ± 1.2	71 [62;81]	4.7 ± 1.7	85 [67;103]	0.1 ±1.8	0.711	0.30	0.068
Vitamine B6 [mg]	1.8 ± 0.4	132 [117;146]	1.5 ± 0.4	117 [102;132]	-0.3 ± 0.4	0.016	1.6 ± 0.3	127 [113;141]	1.6 ± 0.4	131 [116;145]	0.0 ± 0.3	0.913	0.46	0.005
Vitamine B7, Biotin [µg]	52.3 ± 16.3	118 [102;135]	48.5 ± 14.8	109 [93;125]	-3.9 ± 20.7	0.586	46.3 ± 11.7	97 [84;110]	49.5 ± 15.2	116 [98;134]	3.2 ± 15.6	0.349	0.17	0.288
Vitamine B9, Folate [µg]	350.9 ± 109.1	115 [98;131]	310.9 ± 70.6	109 [99;118]	-40.1 ± 97.5	0.184	291.6 ± 94.3	94 [79;109]	292.1 ± 75.9	103 [88;118]	0.5 ± 82.1	0.616	0.19	0.236
Vitamine B12 [µg]	5.7 ± 2.4	112 [75;148]	1.0 ± 1.2	28 [11;45]	-4.6 ± 2.9	<0.001	3.9 ± 1.7	130 [105;155]	4.0 ± 2.0	129 [99;159]	0.1 ± 2.3	0.983	0.73	<0.001
Vitamine C [mg]	125.5 ± 54.3	144 [120;167]	144.1 ± 84.6	160 [121;200]	18.6 ± 101.3	0.42	157.1 ± 80.1	175 [135;214]	126.4 ± 47.3	127 [104;150]	-30.7 ± 72.1	0.043	0.25	0.121
Vitamine D [µg]	3.8 ± 3.2	17 [10;24]	1.7 ± 1.5	15 [2;29]	-2.1 ± 3.7	0.031	3.9 ± 6.3	12 [8;15]	2.7 ± 1.5	28 [-1;58]	-1.2 ± 6.2	0.983	0.24	0.136
Vitamine E [mg]	16.8 ± 6.0	155 [134;177]	19.8 ± 5.4	163 [141;184]	3.0 ± 8.0	0.184	16.1 ± 7.7	142 [111;172]	18.0 ± 8.0	152 [119;185]	1.9 ± 11.0	0.5	0.09	0.574
Vitamine K [µg]	195.6 ± 193.3	254 [143;366]	152.2 ± 131.3	214 [117;310]	-43.4 ± 227.0	0.557	161.4 ± 108.5	246 [172;320]	190.2 ± 152.7	269 [162;376]	28.8 ± 114.7	0.446	0.16	0.316
**Minerals**														
Sodium [mg]	2753.5 ± 822.2	109 [90;128]	1620.7 ± 870.7	81 [60;101]	-1132.8 ± 1202.1	0.004	1861.1 ± 863.5	90 [69;110]	2147.6 ± 820.4	119 [88;149]	286.5 ± 800.2	0.286	0.61	<0.001
Chloride [mg]	4170.7 ± 1222.8	113 [94;132]	2563.5 ± 1298.8	86 [66;106]	-1607.3 ± 1754.9	0.003	2814.6 ± 1189.6	91 [72;110]	3291.0 ± 1150.5	117 [93;141]	476.4 ± 1387.2	0.231	0.58	<0.001
Potassium [mg]	3402.8 ± 651.3	160 [145;175]	2970.9 ± 655.5	144 [126;162]	-431.9 ± 670.2	0.02	3001.4 ± 627.3	144 [126;161]	3046.4 ± 581.7	149 [134;164]	45.1 ± 513.7	0.616	0.38	0.021
Magnesium [mg]	498.1 ± 401.7	145 [83;208]	428.3 ± 107.4	131 [111;152]	-69.9 ± 408.8	0.557	348.4 ± 117.6	110 [91;129]	362.4 ± 99.6	127 [108;145]	14.1 ± 94.8	0.349	0.06	0.715
Zinc [mg]	11.9 ± 2.6	137 [114;160]	8.9 ± 2.2	117 [102;131]	-2.9 ± 3.1	0.002	9.6 ± 2.5	132 [116;148]	10.6 ± 3.6	143 [123;164]	1.0 ± 3.3	0.215	0.55	0.001
Copper [µg]	2260.1 ± 720.2	181 [154;207]	2372.7 ± 603.8	180 [152;208]	112.6 ± 734.7	0.231	1896.4 ± 658.7	146 [119;173]	1931.5 ± 613.2	163 [136;189]	35.1 ± 598.2	0.679	0.05	0.738
Sulfur [mg]	971.0 ± 243.1	---	566.6 ± 131.5	---	-404.4 ± 238.8	<0.001	781.6 ± 191.6	---	790.5 ± 193.5	---	8.9 ± 194.2	0.811	0.75	<0.001
Phosphorus [mg]	1453.4 ± 258.4	187 [170;204]	1127.2 ± 303.5	156 [133;179]	-326.2 ± 210.5	<0.001	1210.0 ± 246.9	165 [146;185]	1289.6 ± 331.4	187 [166;209]	79.6 ± 311.2	0.215	0.64	<0.001
Fluoride [µg]	2004.3 ± 4179.9	45 [-17;108]	953.0 ± 501.6	31 [22;41]	-1051.3 ± 4202.8	0.396	789.0 ± 377.2	24 [18;30]	872.4 ± 435.6	58 [-1;116]	83.5 ± 351.9	0.133	0.24	0.136
Calcium [µg]	1174.5 ± 1412.9	89 [24;154]	551.4 ± 188.7	56 [44;68]	-623.1 ± 1419.1	<0.001	708.4 ± 203.7	67 [56;78]	849.8 ± 260.5	101 [68;135]	141.4 ± 316.6	0.071	0.68	<0.001
Iron [mg]	14.3 ± 3.8	138 [119;157]	13.8 ± 3.7	132 [113;151]	-0.5 ± 4.0	0.948	13.5 ± 4.0	124 [103;144]	13.6 ± 4.1	141 [118;163]	0.1 ± 3.9	0.777	0.06	0.727
Iodine [µg]	121.9 ± 77.2	51 [31;71]	54.7 ± 25.5	32 [23;40]	-67.2 ± 81.4	<0.001	91.9 ± 55.1	43 [34;52]	92.3 ± 28.0	66 [33;100]	0.4 ± 62.7	0.327	0.66	<0.001
Manganese [µg]	5402.7 ± 2411.8	194 [162;226]	8038.3 ± 3563.5	243 [200;287]	2635.7 ± 2617.2	0.001	5630.7 ± 2979.4	155 [114;196]	6324.4 ± 3642.3	218 [142;293]	693.7 ± 2123.9	0.199	0.38	0.019

Data results from three-day weighed food records analyzed with NutriGuide software, including the German Nutrient Data Base (German: Bundeslebensmittelschlüssel)

[a] Nutrient intake is presented as mean ± SD and compared within the groups with the Wilcoxon signed rank test for paired samples.

[b] Treatment effect and p-value between groups was determined using the Mann-Withney-U test, comparing the delta of the nutrient intake (= intake at baseline vs. intake after 8 weeks).

[c] The adequate nutrient supply is depicted as mean [95% confidence interval]. It was calculated as a percentage of the daily recommended intake (DRI) and adjusted to gender and age and under the assumption of moderate movement (Physical Activity Level, PAL 1,6). D-A-CH Reference values are defined by the German (D), Austrian (A) and Swiss (CH) nutrition societies.
